# Antibacterial Activity of Neat Chitosan Powder and Flakes

**DOI:** 10.3390/molecules22010100

**Published:** 2017-01-06

**Authors:** Nury Ardila, France Daigle, Marie-Claude Heuzey, Abdellah Ajji

**Affiliations:** 1 Research Center for High Performance Polymer and Composite Systems (CREPEC), Department of Chemical Engineering, Polytechnique Montréal, P.O. Box 6079, Station Centre-Ville, Montréal, QC H3C 3A7, Canada; nury.ardila@polymtl.ca; 2 Department of Microbiology, Infectiology and Immunology, Pavillon Roger-Gaudry, Université de Montréal, P.O. Box 6128, Station Centre-ville, Montréal, QC H3C 3J7, Canada; france.daigle@umontreal.ca

**Keywords:** chitosan, antibacterial activity, *E. coli*, *L. innocua*, *S. aureus*

## Abstract

This study investigates the antibacterial activity of neat chitosan powder and flakes against three different bacterial species, *Escherichia coli*, *Listeria innocua* and *Staphylococcus aureus*, which are frequent causes of food spoilage. The effect of chitosan concentration and purity, as well as the influence of temperature, ionic strength (salt) and impact of a solid physical support in the medium are examined. Results show that the antibacterial activity of neat chitosan: (i) requires partial solubilisation; (ii) can be promoted by environmental factors such as adequate temperature range, ionic strength and the presence of a solid physical support that may facilitate the attachment of bacteria; (iii) depends on bacterial species, with a sensitivity order of *E. coli* > *L. innocua* > *S. aureus*; and (iv) increases with chitosan concentration, up to a critical point above which this effect decreases. The latter may be due to remaining proteins in chitosan acting as nutrients for bacteria therefore limiting its antibacterial activity. These results on the direct use of chitosan powder and flakes as potential antimicrobial agents for food protection at pH values lower than the chitosan p*K*_a_ (6.2–6.7) are promising.

## 1. Introduction

Chitin is a cellulose-like biopolymer consisting of linear chains of predominantly β-(1 → 4)-2-acetamido-2-deoxy-d-glucose (also named *N*-acetyl-d-glucosamine) residues. Due to its low solubility in organic solvents and low chemical reactivity, chitin is usually transformed into chitosan. Chitosan, the deacetylated form of chitin, is a polysaccharide composed mainly of repeating β-(1 → 4)-2-amino-2-deoxy-d-glucose (or d-glucosamine) units. Several properties of chitosan, including its natural origin, abundance, biodegradability, availability, biocompatibility, mucoadhesivity and reactivity make it attractive in different fields (biomedical, food industry, cosmetology, water purification, among others) [1,2,3]. In particular, its antimicrobial properties [4,5,6] along with its non-toxicity [7] make it of great interest in the food protection area [8,9,10,11,12]. 

Although inherent, the antimicrobial properties of chitosan are affected by different factors. Kong, Chen, Xing and Park [4] have classified most of them into four different types, namely: (1) microbial factors, including microorganism species and cell age; (2) environmental factors such as pH, ionic strength of the medium, temperature and reaction time; (3) intrinsic characteristic of chitosan, such as positive charge density (associated with degree of deacetylation, DDA), molecular weight (MW), chelating capacity, hydrophobic/hydrophilic characteristics and (4) physical state, specifically solution or solid state. Although different authors have evaluated the effects of some of the aforementioned factors on the antimicrobial activity of chitosan solutions [2,5,6,13,14,15,16,17], films [18,19,20], fibers [21,22,23,24], micro- and nanoparticles [25,26,27,28], to our knowledge no study has reported the inhibitory effect of chitosan in a neat discontinuous solid state, such as powder and flakes, nor on its mode of action. A direct usage of chitosan in these forms may be of industrial interest because no processing step is involved. Moreover, a systematic study of chitosan in this state is necessary for a deeper understanding of its antibacterial (AB) action and to broaden its activity and applicability. For example, processed discontinuous solid forms of chitosan such as micro- and nanobeads may be of significant interest in the development of new chitosan-based food packaging materials, but this is beyond the scope of the present work. On the other hand, the mode of antimicrobial action of chitosan powder and flakes may be considered different from the one for chitosan nanoparticles, given the special character of the latter such as their small size and high surface area, as well as the possibility to enter the cells through perfusion [26,29].

Furthermore, while factors such as pH, medium type, bacterial species, and chitosan concentration, MW and DDA have been widely studied for chitosan in solution [2,5,6,13,14,16,17], others such as temperature, ionic strength, the presence of a solid physical support and chitosan purity have received less attention. From the aforementioned factors, temperature, salt concentration (ionic strength) and bacterial species are known to be the most critical in food spoilage and the most relevant in food preservation, but have not been thoroughly considered altogether in chitosan-related studies and therefore are investigated in the present study. Apart from the above, humidity, which is known to be one of the major factors deteriorating the properties of food and accelerating the formation of undesirable organisms, will not be considered in the current research but in future investigations.

In this work, we examine the effect of different critical factors affecting food spoilage and preservation on the AB activity of chitosan in a neat discontinuous solid state (powder and flake-like forms), and under carefully controlled experimental conditions. More specifically, the influence of environmental and microbial factors such as temperature, ionic strength, the presence of a solid physical support, and bacterial species are investigated. Moreover, the effects of chitosan concentration and purity are analyzed. The results show that chitosan in powder and flake form exhibit a high antibacterial activity under conditions close to those of found in contaminated food products. Nonetheless, this activity can be affected positively or negatively by factors such as temperature, ionic strength, chitosan concentration, purity and bacterial species.

## 2. Results and Discussion

Table 1 presents the DDA, MW, polydispersity (PDI), moisture, ash and protein content as well as particle size values of the chitosan (CS) powder (P) and flakes (F) used. Chitosan flakes and powder have a DDA of 90% and 95%, respectively. In average, samples contain 9 wt/v % of moisture and low ash content (0.05%). In addition, chitosan flakes and powder include 8.8 and 176 mg of proteins per gram of chitosan, respectively. According to the suppliers both grades come from the same source (shrimp shells), hence differences in purity may be related to the conditions of the chemical treatment when transforming chitin into chitosan, including the sequence for deproteinization, decalcification and deacetylation, the concentration of the chemicals used and the soaking time [30,31].

**Figure 1:**
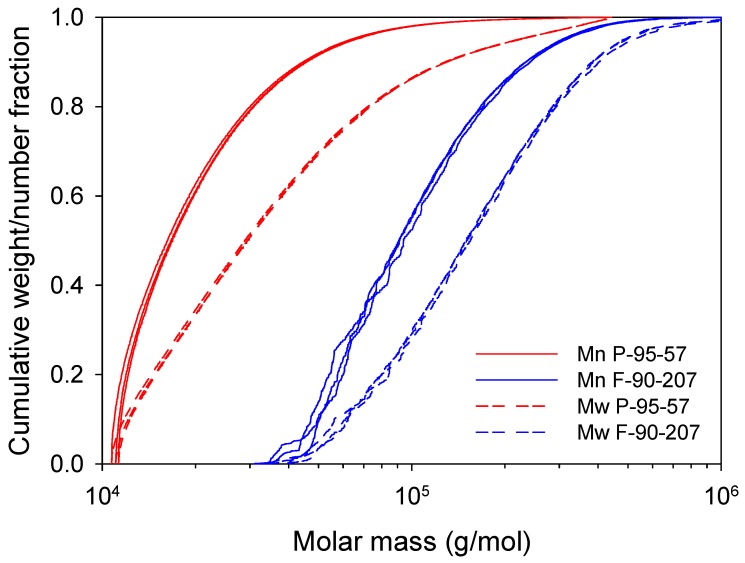
Cumulative weight (Mw)/number (Mn) fraction as a function of molar mass for chitosan powder and flakes.

Regarding the MW and PDI, samples exhibit a weight average MW of 207 and 57 kDa and polydispersity indices of 1.7 and 2.2, respectively, which may also be related to different conditions during chemical treatment. Figure 1 presents the cumulative weight and number fraction as function of molar mass for chitosan. Chitosan flakes (F-90-207) have a narrower size distribution than chitosan powder (P-95-57). In addition, about 5% of chitosan flakes show a molecular weight between 30 to 50 kDa, whilst about 10% of the chitosan powder has a molecular weight near 10 kDa, which may indicate the presence of chitooligosaccharides.

### 2.1. SEM

#### 2.1.1. Elemental Analysis

Table 2 presents the elemental analysis of chitosan. In addition to carbon, nitrogen and oxygen, chitosan samples (both powder and flakes) contain traces (in ppm) of sodium, calcium, chlorine, cobalt and magnesium, probably remaining from chitin and the different stages of the extraction and purification processes [32].

#### 2.1.2. Particle Size, Thickness, Shape and Particle Size Distribution 

**Figure 2:**
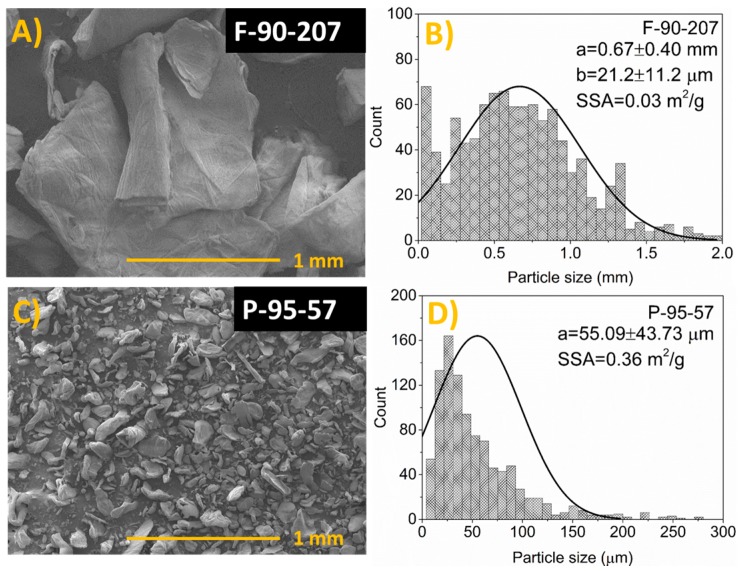
Chitosan in flakes and powder form (**A**,**C**) and their particle size distribution (**B**,**D**). The symbols a, b and SSA (in **B** and **D**) represent the average particle size, thickness and the specific surface area, respectively.

Figure 2 presents SEM micrographs of the chitosan samples. Their average particle size and particle size distribution are shown in Table 1 and Figure 2, respectively. According to the micrographs, chitosan flakes, which are of the order of millimeter size (0.7 mm in average), present a thickness of 21.2 μm, an irregular shape, and higher particle size values and broader particle size distribution than the powder grade. This is probably the result of the high variability and asymmetry of their dimensions. In the case of powder, distribution is right-skewed and particles are of micrometer size (55 μm, in average). Considering both chitosan in flakes and powder as spheres having a bulk density of 0.3 g/cm^3^ [33], the specific surface area varies from 0.03 (flakes) to 0.36 (powder) m^2^/g (Figure 2). The specific surface area is considered an important factor for the antibacterial activity.

### 2.2. Antibacterial Assays

#### 2.2.1. Effect of Chitosan Concentration

**Figure 3:**
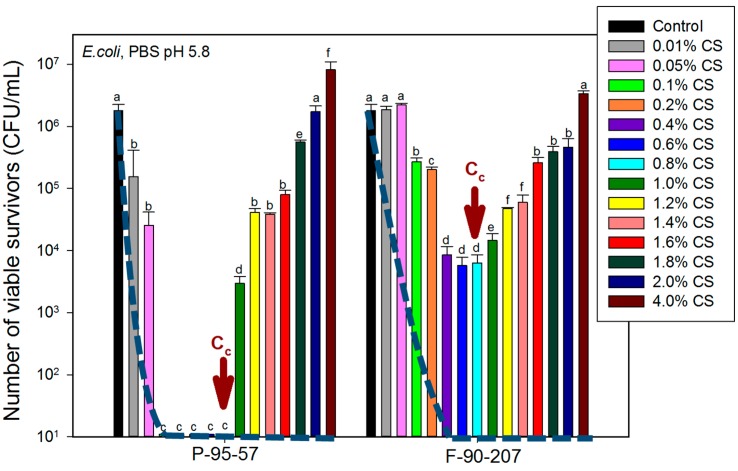
Effect of chitosan concentration in PBS on the number of viable survivors. C_c_ is the critical concentration above which the AB activity of chitosan decreases. Dashed lines represent the reduction in bacterial concentration after deproteinization. Samples are P-95-57 (powder) and F-90-207 (flakes). The number of viable organisms was the same after 18 and 48 h incubation on the agar plates, suggesting that recovery from sub-lethal injury had not taken place. For each chitosan grade, means that do not share a letter are significantly different with a confidence level of 95% by Tukey Pairwise Comparisons.

Figure 3 shows the effect of chitosan concentration for the two chitosan grades, when suspended in PBS. According to the results, the AB activity of chitosan increases with concentration up to a certain value, named the critical concentration, C_C_, after which this activity decreases. The C_C_ was found to be between 0.4 and 1.2 wt/v % without any apparent pattern regarding DDA, MW, bacterial species or medium.

It is noteworthy that: (i) the pH of the medium varied between 5.8 and 7.0 after the incorporation of chitosan (0.01%–0.5% pH 5.8; 0.5%–1.0% pH 6.2; 1.0%–2.0% pH 6.5 and 2.0%–4.0% pH 7.0) to the PBS medium (pH 5.8), which is mainly related to the increase of protonated chitosan amino groups (NH_3_^+^) and (ii) bacterial population in the control remained invariable in this pH range. 

**Figure 4:**
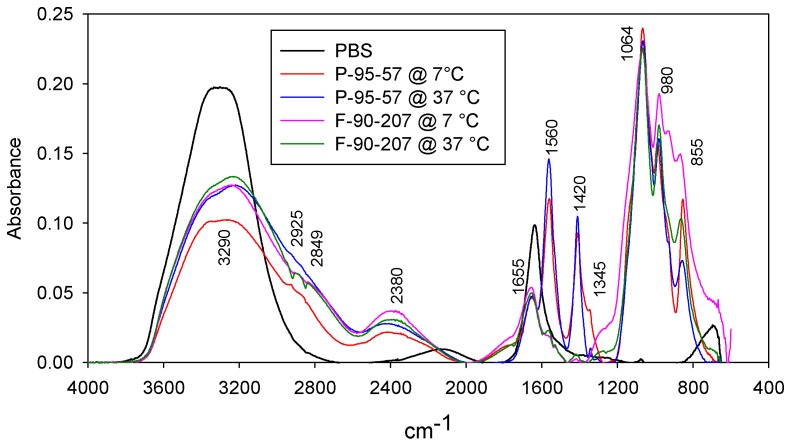
FTIR spectra: Peaks at 1345, 1420, 1560, 1655 and 3290 cm^−1^ confirm the solubility of chitosan powder and flakes in the suspensions during the AB tests.

Protonation may also cause partial solubility of chitosan suspensions, given the pH of the medium. The potential solubility of chitosan powder and flakes during the AB tests was verified qualitatively by ATR spectroscopy, at a chitosan concentration of 0.4 wt/v %. Figure 4 compares the FTIR spectra of PBS with the filtrate of chitosan suspensions subjected to the same conditions as in the AB tests at 7 and 37 °C. Solubility of chitosan suspensions was confirmed given the identification of the main characteristic peaks of chitosan at 1345, 1420, 1560, 1655 and 3290 cm^−1^, which correspond to a CO–NH deformation and to CH_2_ group (amide III); C-H_2_ stretching bending; amide II band and the N–H stretching of amine II; CONH_2_ group and stretching of C=O (amide I); and –OH and –NH stretch, respectively. In particular, the amide I bands at 1655 cm^−1^ or the amide II band at 1560 cm^−1^ are identified as characteristic *N*-acetylation bands associated with amine and amide groups [34,35].

Preliminary AB tests showed that chitosan powder and flakes were not active at pH values higher than the chitosan p*K*_a_ (6.2–6.5) [36,37], and indicated the need for a partially solubilized state to exert any AB effect. This point highlights the main difference with chitosan micro- and nanoparticles, in which the AB action of chitosan could be achieved at acidic and neutral pH values [25,26,28].

**Figure 5:**
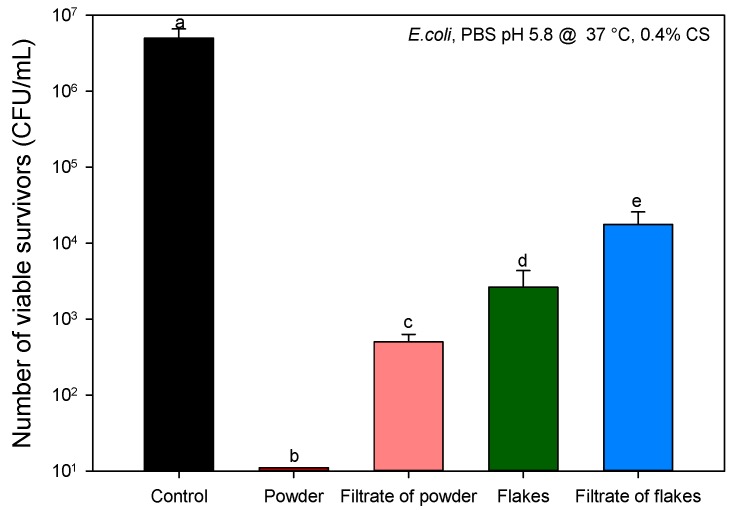
Recovery of viable bacteria after exposure of chitosan and filtrate from chitosan suspensions to *E. coli*. The number of viable organisms was the same after 18 and 48 h incubation on the agar plates, suggesting that recovery from sub-lethal injury had not taken place. Means that do not share a letter are significantly different with a confidence level of 95% by Tukey pairwise comparisons.

The contributions to the AB activity from the partially solubilized chitosan (filtrate subjected previously to the same conditions as in the AB tests) and from the solid state particles were quantified. Figure 5 presents the decrease in bacterial density after exposure of *E. coli* to 0.4 wt/v % chitosan and to the filtrate from chitosan suspensions. AB results allowed quantifying a reduction of 4.0 and 2.5 log in bacterial density from the chitosan solubilized in the suspensions with powder and flakes, respectively. An additional contribution of 2.7 and 0.8 logs reduction to the AB activity was obtained by the presence of the chitosan powder and flakes, which may act as physical supports for the attachment of bacteria, as previously reported for the case of chitosan microspheres [27]. Hence, these results suggest that the presence of a solid form may favor chitosan AB activity. In this regard, AB assays were performed in the presence of calcium carbonate particles (CaCO_3_), a chemical compound lacking intrinsic AB activity.

**Figure 6:**
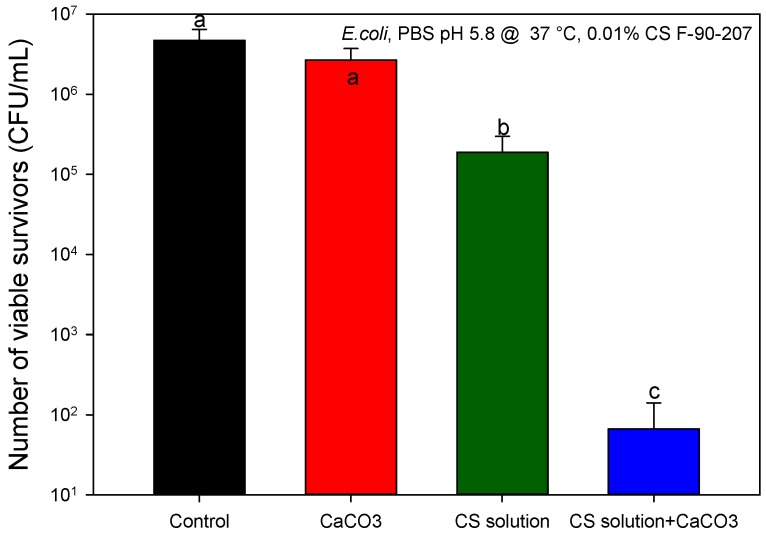
Recovery of viable bacteria after exposure of CaCO_3_ and CS solution (F-90-207) to *E. coli*. The number of viable organisms was the same after 18 and 48 h incubation on the agar plates, suggesting that recovery from sub-lethal injury had not taken place. Means that do not share a letter are significantly different with a confidence level of 95% by Tukey pairwise comparisons.

Figure 6 shows the surviving bacteria after exposure of *E. coli* to CS solution and CaCO_3_. At a concentration of 0.01 wt/v %, CS solution reduces bacterial density by 1.4 log. However, *E. coli* reduction increases up to 4.8 logs when CaCO_3_ is present in the medium. As CaCO_3_ did not display AB activity in the absence of chitosan, these results indicate that the presence of a solid particles enhances the AB activity of the CS solution filtrate and may facilitate the attachment of bacteria. Similarly, chitosan powder and flakes may serve as physical solid supports to enhance the AB activity of the partially solubilized chitosan. Attachment of bacteria to chitosan is due to electrostatic interactions between the positively charged chitosan with the negatively charged cell surface. As CaCO_3_ reacts in the presence of a means acid (given the HCl added to the medium when adjusting the pH to 5.8) producing Ca^2+^, hence it is believed that bacteria may attach to calcium when seeking nutrients for microbial growth [38].

Based on the previously discussed analyses, two hypotheses are considered to explain the existence of a critical chitosan concentration, as observed in Figure 3. The first one considers that a possible agglomeration of particles at the bottom of the assay tubes leaves less chitosan solubilized and fewer particles in contact with bacteria, thereby decreasing the AB activity of chitosan. However, AB tests conducted in Erlenmeyer flasks with higher surface area (approx. 16–25 times) than in assay tubes yielded the same trend than the ones presented in Figure 3, and consequently this hypothesis was rejected. The second hypothesis considerers that impurities, such as minerals and proteins remaining in chitosan [39] or chitosan itself may represent a nutrient source for bacteria and therefore be responsible for the decrease in the AB activity above the critical concentration. PBS buffer medium contains no nutrients for bacteria and at 4 wt/v % chitosan, bacterial density increases over the control and at a higher bacterial growth rate in chitosan powder than flakes, which is congruent with the higher protein content (Table 1). This allows speculating that proteins act as nutrients for bacteria. Hence, a deproteinization step via enzymatic activity for these two chitosan grades was carried out, and the AB results are shown as dashed lines in Figure 3. After the removal of proteins, the AB activity of chitosan increases and lower concentrations are sufficient to eradicate bacteria. In addition, by increasing chitosan content, the AB activity increases up to a certain concentration and remains even if the concentration is further increased. Accordingly, impurities in solid state chitosan such as proteins can limit its AB efficacy. On the other hand, the low ash values reported in Table 1 discard a feeding effect from minerals. Chitosan itself (in the form of chito-oligosaccharides observed in Figure 1) as a nutrient source for bacteria was also discarded since at 4 wt/v % bacterial density was totally reduced only for deproteinized chitosan, while chito-oligosaccharides may be present before and after enzymatic treatment.

#### 2.2.2. Identification of Proteins

**Figure 7:**
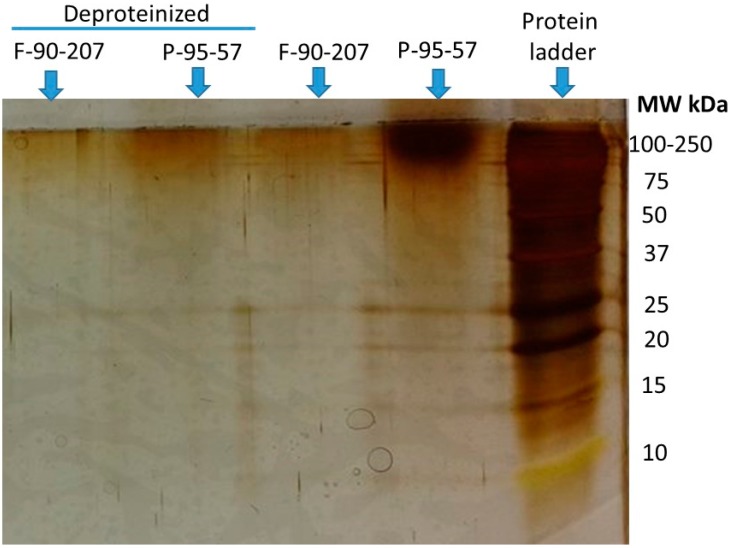
Identification of proteins before and after deproteinization of chitosan. Samples are P-95-57 (powder) and F-90-207 (flakes).

Figure 7 shows a SDS-PAGE electrophoresis test result for the identification of the proteins remaining in the two chitosan samples, before and after deproteinization. High molecular weight proteins, in the range of 100–250 kDa, were detected in chitosan before the deproteinization step. Those are indicated as intense bands in Figure 7. Once proteins are removed, a decrease in the intensity of the bands is observed for both chitosan grades. This also confirms that proteins are mostly removed after the enzymatic treatment.

#### 2.2.3. Effect of Temperature 

Temperature is an important parameter to consider when seeking practical applications such as in the food packaging sector. Generally, in vitro AB tests of chitosan in solution form are carried out under optimal conditions for the growth and survival of bacteria, such as 37 °C in the case of *E. coli.* To our knowledge, the effect of temperature on the AB efficacy of chitosan in solution has barely been examined [40,41], not to mention in a discontinuous solid form.

**Figure 8:**
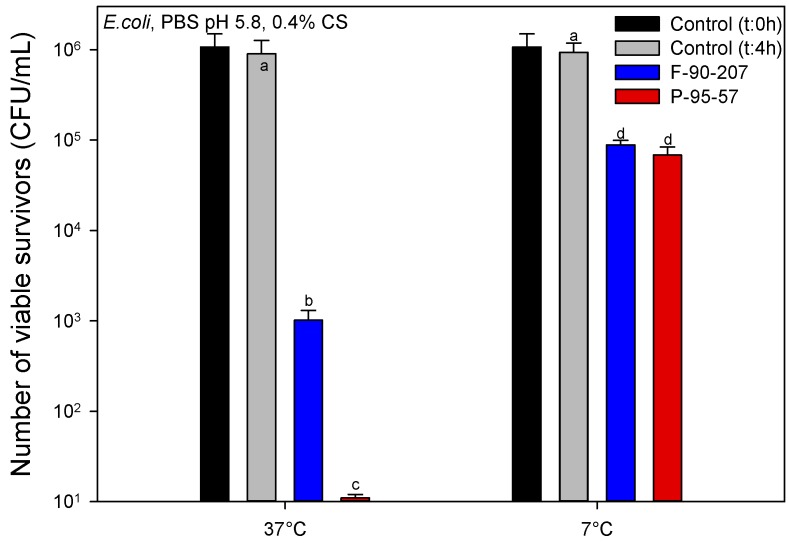
Effect of temperature on the antibacterial activity of chitosan (number of viable survivors). Bars with different letter are significantly different (*p* < 0.05). Samples are F-90-207 (flakes) and P-95-57 (powder). The number of viable organisms was the same after 18 and 48 h incubation on the agar plates, suggesting that recovery from sub-lethal injury had not taken place. Means that do not share a letter are significantly different with a confidence level of 95% by Tukey pairwise comparisons.

Figure 8 shows the effectiveness of chitosan at 7 ± 1 °C and 37 ± 1 °C and pH of 5.8. These values correspond to temperatures close to those of refrigerated food products and optimal bacterial growth, respectively. As presented in Figure 8, the AB activity of chitosan highly depends on the incubation temperature, with a noticeably greater AB efficacy at 37 °C. At this temperature, a total inhibition of bacterial density for the powder chitosan grade, and a decrease in 3 log CFU/mL (approx. 99.9% of bacteria) for the flakes, are observed. By contrast, despite the fact that the AB activity strongly decreases at 7 °C, as shown in Figure 8, both chitosan grades reduce bacterial density by more than 1 log CFU/mL (approx. 90% of bacteria). Hence, notwithstanding the limiting effect of temperature and the fact that total inhibition of bacteria was not achieved, results are still promising regarding chitosan AB activity in refrigerated conditions. Similar results were reported recently in which a higher susceptibility of bacteria to the action of chitosan nanoparticles in high temperature conditions was found [25].

Different factors may contribute to the stronger activity at 37 °C seen in both chitosan grades. First, as temperature controls the acid dissociation constant K_a_ (p*K*_a_ = −log *K*_a_) [42], an increase in the temperature favors the AB activity since *K*_a_ increases. In this work, the p*K*_a_ of chitosan powder and flakes was determined by titration [43] at 0 °C and 37 °C. The p*K*_a_ decreases from 6.7 to 6.6 for chitosan flakes, and from 6.6 to 6.2 for chitosan powder when the temperature increased from 0 to 37 °C, respectively. The smaller the value of p*K*_a_, the larger the extent of dissociation and the number of protonated amino groups, which should favor the AB activity at higher temperature. 

Second, a higher chitosan solubility and chain mobility may be achieved at 37 °C than at 7 °C. Figure 4 illustrated the potential solubility of chitosan powder and flakes at these temperatures. The higher AB activity of chitosan powder with respect to chitosan flakes at 37 °C may be accounted for by a lower particle size, lower MW and higher DDA content, which could favor its solubility, given the pH of the medium (5.8). In addition, chitosan powder has a higher content of low MW species (chito-oligosaccharides) as shown in Figure 1, which may have contributed to the higher AB effect [44].

Finally, Tsai and Su [40] have suggested that low temperature may induce changes in the bacterial cell structure by decreasing the number of binding sites on the surface (or electronegativity). Consequently, less protonated chitosan amino groups may interact with the available negatively charged sites in the bacteria surface, resulting in a decreased chitosan AB activity. According to our results, the AB activity for both chitosan grades is highly reduced when the temperature decreases but without significant difference regarding the efficacy between the two grades (*p* > 0.05), as observed at 37 °C (*p* < 0.05). Therefore, it is speculated that this mechanism (decrease of number of binding sites) may influence the most and strongly limits the AB efficacy, regardless of the chitosan grade. 

#### 2.2.4. Effect of Salt Concentration and Ionic Strength

**Figure 9:**
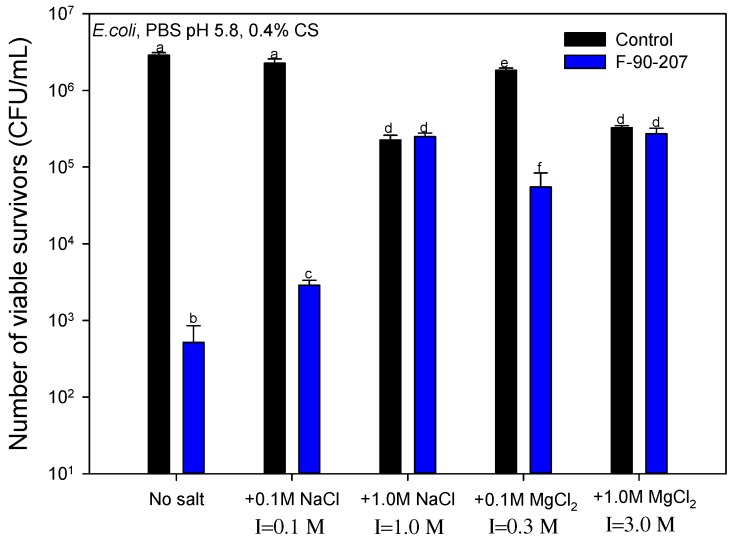
Effect of salt concentration and ionic strength (I) on the antibacterial activity of chitosan. Bars with different letters are significantly different (*p* < 0.05). Sample is F-90-207 (flakes). The number of viable organisms was the same after 18 and 48 h incubation on the agar plates, suggesting that recovery from sub-lethal injury had not taken place. Means that do not share a letter are significantly different with a confidence level of 95% by Tukey pairwise comparisons.

Salts are commonly incorporated into food as additives and preservatives. Their presence can favor the chelating capacity of chitosan for metal ions and consequently compromise its antibacterial properties. Figure 9 shows the effect of salt concentration and ionic strength (I) on the AB activity of chitosan flakes. As the concentration of NaCl and MgCl_2_ increases from 0.1 M to 1.0 M, the AB activity of chitosan decreases. This effect can be explained through two mechanisms and the Debye-Hückel equation [4,45,46,47]. First, given the acidic conditions of the medium (pH 5.8), protonated chitosan amino groups may trigger electrostatic attraction of anionic compounds, including metal anions [48]. Consequently, the interaction of chitosan with metal ions in the aqueous medium leaves less chitosan amino groups available for contact with bacteria. Secondly, the negatively charged components (lipopolysaccharides and proteins) on the Gram-negative *E. coli* bacteria surface may interact with the existing cations in the medium instead of interacting with chitosan, consequently lowering the apparent AB activity. This interaction certainly occur given bacteria adsorb essential nutrients such as Ca^2+^ for microbial growth [38]. Otherwise, as electrolytes are dispersed in the medium, the electrostatic interactions of chitosan may be screened by the free charges, causing a net “double-layer” interaction decay with a characteristic length known as Debye-length $\kappa^{-1}$ [49]. For an electrolyte:

(1) $$\kappa^{-1}=\left[(\varepsilon_{r}\varepsilon_{0}k_{B}T\right)/\left(2N_{A}e^{2}I\right){]}^{1/2}$$

where $\;\varepsilon_{r},\varepsilon_{0},k_{B},T,N_{A},e\;\mathrm{and}\;I\;$ are the dielectric constant, permittivity of the free space, Boltzmann constant, absolute temperature, Avogadro number, elementary charge and ionic strength in the medium, respectively [47]. Since all previous parameters are the same in our study except for the ionic strength, $\kappa^{-1}$ can be simplified as $\kappa^{-1}=KI^{-1/2}$ nm, where *K* is a constant. Accordingly, the Debye-length decreases with increasing ionic strength (or salt content in this case), explaining the decrease of the AB activity when NaCl or MgCl_2_ are added. This is a consequence of a decrease of the electrostatic repulsions in chitosan (screening of the positively charged chitosan amino groups), limiting the interactions with the negatively charged bacterial surface. 

In addition, our results show that the type of salt (ions) in the medium also influences the AB effectiveness of chitosan. This effect can be observed when analyzing the two types of salt at a concentration of 0.1 M. According to Figure 9, the AB efficacy of chitosan is weaker when Mg^2+^ ions are present in the medium in comparison with Na^+^, i.e., the presence of Na^+^ ions is less detrimental to the AB efficacy of chitosan. At 0.1 M, the ionic strength in the medium is three times higher for MgCl_2_ than for NaCl, which implies a decrease of about 1.7 times·$\kappa^{-1}$. Consequently, more charges are screened, limiting more significantly the AB activity. The same explanation is valid when comparing the AB efficacy of chitosan at 1.0 M NaCl (I=1.0 M, $\kappa^{-1}=1\;K$·nm) with 0.1 M MgCl_2_ (I=0.3 M, $\kappa^{-1}=1.82\;K$·nm). 

On the other hand, when a high concentration of salt is used (1.0 M), the inhibitory effect of chitosan fades more in comparison with 0.1 M, but by an equal amount for both salts (*p* > 0.05). Although the Debye-length theory is only valid at low concentrations and breaks down when the ionic strength is over 0.1 M [50,51], a rough approximation indicates that at 1.0 M, $\kappa^{-1}$ is 1.0 *K* nm and 0.6 *K* nm for NaCl and MgCl_2_, respectively. In this case, despite the Debye-length is meaningfully different, no significant difference in the drop of the AB efficacy is observed, inferring that chitosan charges are totally screened. However, the concentration of salt is high enough to limit entirely the AB activity of chitosan as well as killing bacteria, as observed in the control samples containing no chitosan in Figure 9. The death of *E. coli* cells is probably the result of their effort to reduce the concentration gradient of salt inside and outside the cell walls, causing morphological damage and loss of cellular integrity, given such a high salinity content [52].

#### 2.2.5. Influence of Bacterial Species

Table 3 presents the survival and reduction (%) of bacterial density when three different species of bacteria are in contact with chitosan. Chitosan presents a noticeable greater AB activity against *E. coli* in comparison with *L. innocua* and *S. aureus*. For instance, while *E. coli* density is reduced by more than 99.5% by either chitosan powder or flakes, *L. innocua* and *S. aureus* are more sensitive to the effect of chitosan in powder form.

**Figure 10:**
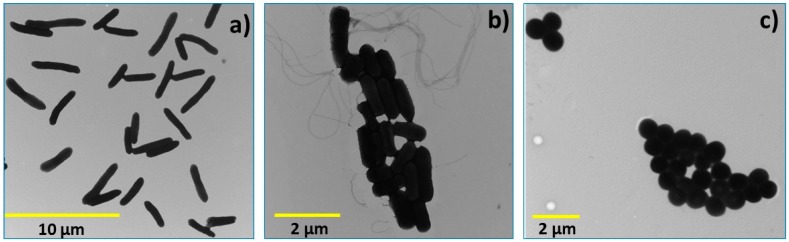
Morphology of intact: (**a**) *E. coli*; (**b**) *L. innocua*; (**c**) *S. aureus* cells. Images were kindly provided by Mounia Arkoun from the Chemical Engineering Department, Polytechnique Montréal.

Figure 10 shows the morphology of *E. coli*, *L. innocua* and *S. aureus* cells via TEM. *E. coli* and *L. innocua* cells shows typically rod-shaped forms of 3.41 ± 0.68 and 1.22 ± 0.20 μm in length, and 1.01 ± 0.2 and 0.53 ± 0.05 μm in height, respectively. However, *S. aureus* cells are spherical, with a diameter of 0.81 ± 0.16 μm. Those dimensions allow speculating that discontinuous solid state chitosan may interact more easily with *E. coli* rather than with *L. innocua* and *S. aureus* cells, which falls within the same sensitivity order seen in our findings. However, our recent study on chitosan nanoparticles demonstrated that the AB activity is independent of the size and form of the cells [25].

Some studies on chitosan solutions have reported a higher sensitivity against Gram-negative species [2,5,53,54]. The higher sensitivity found in the case of *E. coli* (Gram-negative) in comparison with *L. innocua* and *S. aureus* (both Gram-positive) can be explained first in terms of the differences in the cell surface characteristics between the Gram types, such as hydrophilicity, negative charge density and adsorptive properties. A generally stronger net negative charge in the Gram-negative strains [55,56,57] may favor larger electrostatic interactions between the positively charged chitosan amino groups and the negatively charged bacteria surface. Other factors such as higher hydrophilicity and adsorption of chitosan on cell wall in Gram-negative strains, with respect to Gram-positives ones, could increase the AB effect [56]. In addition to the above, the structural organization in the envelope/membrane constituents of Gram positive and Gram negative strains may play the most important role influencing the AB activity. Both strains have similar composition regarding phospholipids, glycoproteins, cholesterol and polysaccharides. However, the way they are placed and organized vary between the strains. For instance, Gram positive consist of a single phospholipid layered membrane and a thick murein (peptidoglycan), while Gram negative consist of a single phospholipid layered membrane a thinner peptidoglycan layer and covered by a phospholipidic bilayered membrane. The smaller thickness of the peptidoglycan layer in Gram-negative strains (7 to 8 nm), in comparison to the Gram-positive strains (20 to 80 nm), may render it more sensitive to the action of chitosan [58].

However, despite the above, many reports have demonstrated higher AB activity of chitosan in solution form against *S. aureus* in comparison with *E. coli* [6,14,15,58], which is opposite to the findings of our current work. In those cases, authors explained the higher AB effect on the Gram-positive strains (such as *S. aureus*) as a consequence of the absence of the outer membrane barrier in comparison with the Gram-negative strains (such as *E. coli*) [14]. On the other hand, experimental data provided by Tsai et al. [59] allow to infer that the sensitivity of bacteria to chitosan is not dependent on the Gram-type (Gram-positive or Gram-negative) nor dependent on the bacterial species, but dependent on the strain. This would explain the controversial findings amongst different authors when comparing the effectiveness of chitosan.

Our results demonstrated that chitosan needs at least partial solubilisation for an AB effect. Thereby, the lower MW (which include the presence of low MW species or chito-oligosaccharides, even in small quantities) and the higher DDA favors the solubility of chitosan powder and its AB activity. Further research should be performed in order to quantify the solubility of each chitosan grade.

Owing to the size and shape of discontinuous solid state chitosan, it is considered that in addition to solubilisation, the AB action requires a direct contact between chitosan and the cell surface, with a probable microbial cell adsorption not only onto the surface of chitosan powder and flakes but also on the surface of CaCO_3_ particles, which notably enhances the AB activity. However, having only chitosan particles will avoid the need of an additional solid support for optimum AB activity. Other studies have demonstrated the adsorption properties of chitosan powder and flakes to residues and for removal of metals [48,60]. The higher sensitivity of bacteria to powder chitosan might be due to the larger specific surface area and the closer similarity of its size order with the cells, when compared to chitosan flakes. It has been reported that lowering chitosan particle size improves the antibacterial activity [25]. Other properties such as higher lead sorption capacity [61] and higher cytotoxicity towards tumor cells [62] have also been reported as improving with decreasing particle size.

The mode of AB action may differ from that reported for chitosan nanoparticles [25], since the AB activity of powder and flakes require acidic pH and their sizes prevent them from penetrating into cells as compared with nanoparticles. Hence, it is suggested that one part of the AB action is exerted by the direct contact of protonated chitosan powder’s and flakes’ surface with the negatively charged cell wall; and the other, by the solubilized chitosan which may deposit on bacteria surface affecting the cell permeability and leading to the leakage of proteinaceous and other intracellular constituents [27,63]. On the other hand, further research is required in order to evaluate the cytotoxicity of chitosan powder and flakes, which is critical for food packaging and other industrial applications. Different studies have evaluated the cytotoxicity of chitosan nanoparticles [62,64], which can be more critical than chitosan powder and flakes, because they could penetrate the cells through pervasion and alter the DNA and mRNA functions. For instance, Qi et al. [62] reported high cytotoxic activity of chitosan nanoparticles toward tumor cells while low toxicity against normal human liver cells.

## 3. Materials and Methods

### 3.1. Materials

Chitosan (CS) in powder (P) and flake form (F) were obtained from Primex (Siglufjordur, Iceland) and BioLog GmbH (Landsberg, Germany), respectively. They were characterized in terms of DDA, MW, polydispersity (PDI), moisture, ash, protein content and particle size, as presented in

Table 1. Protein bovine serum albumin (BSA)—98% purity—and enzyme proteinase K and Glacial acetic acid were obtained from Sigma Aldrich (Oakville, ON, Canada). Calcium carbonate (CaCO_3_) with a particle size between 3 and 13 μm was obtained from Univar (Surrey, BC, Canada). All other chemicals and reagents were of analytical grade and used without further purification.

#### Bacteria Strains and Culture

Cultures of *Escherichia coli* (*E. coli* strain DH5α, non-pathogen), *Listeria innocua* (*L. innocua* strain ISPQ3284, non-pathogen), *Staphylococcus aureus* (*S. aureus* strain 54–73, pathogen) were obtained from the laboratory of microbiology, infectiology and immunology (Université de Montréal, Montréal, QC, Canada). They were selected as representative bacteria since they are some of the most frequent bacteria found in food spoilage.

### 3.2. Methods

#### 3.2.1. Infrared Spectroscopy (FTIR)

The DDA values were verified and determined (when the company did not provide this information) via FTIR as described in Tsaih and Chen [65]. Samples were prepared in KBr disk form, where KBr disks were compounded from dry mixtures of about 1 mg of chitosan sample and 100 mg of KBr. FTIR spectra were recorded on a Spectrum 65 FT-IR spectrometer (Perkin-Elmer, Woodbridge, ON, Canada) with a resolution of 4 cm^−1^ and 32 accumulations in the wavenumber range of 600 to 4000 cm^−1^. 

#### 3.2.2. Gel Permeation Chromatography (GPC)

The average MW and polydispersity index (PDI) for chitosan samples were determined by size-exclusion chromatography (SEC) as described in Lavertu et al. [66]. Measurements were performed on a Gel Permeation Chromatography (GPC) system consisting of an LC-20AD isocratic pump (Shimadzu, Kyoto, Japan), an autosampler SIL-20AC HT (Shimadzu), an oven CTO-20AC (Shimadzu) coupled with a Dawn HELEOS II multiangle laser light scattering detector (Wyatt Technology Co., Santa Barbara, CA, USA), an Optilab rEX interferometric refractometer (Wyatt Technology Co.), and two Shodex OHpak columns (SB-806M HQ and SB-805 HQ) connected in series. The mobile phase was an acidic aqueous buffer (AcOH 0.15 M, AcONa 0.1 M, NaN_3_ 0.4 mM, 0.1 M NaCl) and a chitosan *dn/dc* value of 0.205 was used (laser’s wavelength of 658 nm).

#### 3.2.3. Thermogravimetric Analysis (TGA)

##### Moisture Content

The moisture content in chitosan powder and flakes was determined according to the AOAC standard methods 930.15 [67] in a thermogravimetric analyzer TGA Q500 from TA Instruments (New Castle, DE, USA). Approximately 10 mg of chitosan were heated from room temperature to 150 °C, at a rate of 10 °C min^−1^ under a nitrogen atmosphere.

##### Ash Content

The ash content in chitosan powder and flakes was characterized according to the AOAC standard methods 942.05 [67] using the same thermogravimetric analyzer. Approximately 10 mg of chitosan were heated from room temperature to 900 °C at a rate of 10 °C·min^−1^ under an air atmosphere.

##### Protein Content

The protein content was determined by ultraviolet (UV) light at 280 nm on a Cary 5000 UV–vis-NIR spectrophotometer (Agilent Technologies, Santa Clara, CA, USA). First, a calibration curve was done with bovine serum albumin (BSA) as standard protein at different concentrations (0.01, 0.05, 0.10, 0.25 and 0.50 wt/v %). Then, the protein content was calculated by correlating the absorbance of each chitosan sample (dissolved in 1 *v*/*v* % HCl) with the corresponding concentration in the calibration curve.

#### 3.2.4. Deproteinization and Identification of Proteins

The deproteinization step was performed using the enzyme proteinase K. In this case, a buffer solution consisting of 30 mM Tris-Cl, 30 mM EDTA, 5% Tween 20, 0.5% Triton X-100 and 800 mM GuHCl was prepared and pH was adjusted to 8.0. Chitosan in powder and flake form was added at a temperature of 50 °C, resulting in suspensions since chitosan is not soluble above its p*K*_a_ (6.2–6.5) [36,37]. Then, proteinase K was added at a concentration of 100 μg·mL^−1^ under shaking during 15 min and finally the temperature was increased to 60 °C to stop the enzyme effect. Deproteinized chitosan was washed, centrifuged and dried at 60 °C overnight.

The determination of proteins molecular weight was done using polyacrylamide gel electrophoresis (PAGE) in the presence of sodium lauryl sulfate (SDS) at a concentration of 15% Tris-HCl. In this case, 40 μL of filtrate from a 4 wt/v % chitosan (before and after deproteinization) suspension in water at pH 7.0 was injected into the gel. Silver staining was used for the recognition of the protein bond.

#### 3.2.5. Scanning Electron Microscopy (SEM)

##### Particle Size

SEM images of chitosan powder and flakes were obtained using a JSM-7600 TFE field emission gun (JEOL, Calgary, AB, Canada) operated at 2 kV. The particle size and thickness were determined using Image-Pro^®^ Plus software (version 5.1 from Media Cybernetics, Rockville, MD, USA) and taking the average value of 1000 particles.

#### 3.2.6. Elemental Analysis

The qualitative determination of chitosan powder and flakes composition was done via Energy Dispersive X-ray spectroscopy (EDS) using a JEOL JSM-840A scanning electron microscope (Oxford Instruments, Abingdon-on-Thames, UK) operating at 20 kV.

#### 3.2.7. Attenuated Total Reflectance Spectroscopy (ATR)

The potential solubility of chitosan powder and flakes during the AB tests was evaluated via ATR. Chitosan suspensions were prepared in the same conditions as for the AB tests, filtrated at room temperature by using Grade 1 Qualitative filter paper (Whatman^TM^ porous size of 11 μm), and then analyzed by placing one droplet of the filtrate directly on the surface of the ATR crystal and left overnight until complete drying before acquiring the spectra. These were recorded on a Perkin-Elmer Spectrum 65 FT-IR spectrometer (Perkin-Elmer, Woodbridge, ON, Canada) with a resolution of 4 cm^−1^ and 32 accumulations in the wavenumber range of 600 to 4000 cm^−1^.

#### 3.2.8. Transmission Electron Microscopy (TEM)

TEM analyses on fresh bacteria were performed according to the method of Arkoun, et al. [63]. Briefly, overnight cultures containing 10^6^ colony forming units per milliliter (CFU/mL) of the selected bacteria were centrifuged (8000 rpm/3 min) and the resulting pellets were resuspended in a 2 *v*/*v* % glutaraldehyde solution (phosphate buffer saline, PBS at pH 7.4) to fix the cells at 4 °C overnight. Then, 10 μL of each sample was deposited on Formvar carbon-coated grids containing one drop of 1% Alcian Blue. Cells were then subjected to 5 min post-fixation with paraformaldehyde (2 *v*/*v* %, PBS) and grids were stained using a drop of filtered 2 *v*/*v* % phosphotungstic acid (PTA, pH 7.0) for 30 s. A series of filtration and/or washing treatment were performed after each step in order to remove excess liquid, fixative or staining. Finally, TEM observation was performed using a CM100 transmission electron microscope (Philips Electron Optics, Eindhoven, The Netherlands) and digital micrographs were captured using an XR80 CCD digital camera (Advanced Microscopy Techniques, Woburn, MA USA).

#### 3.2.9. Antibacterial (AB) Assays

In this study, one Gram-negative (*E. coli*) and two Gram-positive strains (*L. innocua* and *S. aureus*) were used (Figure 10). The microorganisms were grown in a nutritional rich medium (Brain Heart Infusion broth or BHI) under constant agitation for 24 h at 37 °C, in order to reach a density of 10^9^ colony forming units per milliliter (CFU/mL). After 24 h, the bacteria culture were diluted in a buffer, a non-permissive growth condition (phosphate buffered saline or PBS solution), in order to reach a density of approximately 10^6^ CFU/mL. Preliminary AB tests showed that chitosan powder and flakes were not active at pH values higher than chitosan p*K*_a_, and indicated the need of a solution state for the AB activity of chitosan. Therefore, the pH of the PBS solution was altered intentionally to 5.8 (with HCl 1 M). Chitosan powder and flakes were sterilized under UV light for 20 min prior to the preparation of the chitosan suspensions. 

• Effect of Chitosan Concentration

Chitosan suspensions at concentrations between 0.01 and 4 wt/v % were prepared in 5 mL of PBS containing approximately 10^6^ CFU/mL of *E. coli*. Suspensions were incubated during 4 h at 37 ± 1 °C and 22% ± 1% relative humidity (RH) in a shaker. Serial dilutions of the inoculated suspensions were plated on BHI agar (unless otherwise specified) and incubated for 18 h at 37 ± 1 °C and 34% ± 1% RH for the counting of the surviving bacteria (CFU/mL). Plates were verified after 48 h to corroborate that the recovery of viable organisms from sub-lethal injury had not taken place. These dilution and enumeration methods were used for all the other antibacterial following tests described below: 

• Exposure of chitosan and filtrate from chitosan suspensions to *E. coli*

Chitosan suspensions at a concentration of 0.4 wt/v % were prepared in 5 mL of PBS and placed during 4 h at 37 ± 1 °C and 22% ± 1% RH in a shaker. Suspensions were filtrated at room temperature by using Grade 1 Qualitative filter paper (Whatman^TM^ porous size of 11 μm). Then, chitosan suspensions and the filtrate from chitosan suspensions were inoculated with approximately 10^6^ CFU/mL of *E. coli* and incubated during 4 h at 37 ± 1 °C and 22% ± 1% RH in the same shaker.

• Exposure of CaCO_3_ and chitosan solution to *E. coli*

Chitosan solution was prepared by dissolving 1 wt/v % chitosan flakes in 1 *v*/*v* % acetic acid aqueous solution, under magnetic stirring and at room temperature until complete dissolution of the solutes. Chitosan solution was diluted into 5 mL of PBS containing approximately 10^6^ CFU/mL of *E. coli* until reach a concentration of 0.01 wt/v % chitosan. CaCO_3_ suspensions at a concentration of 0.1 wt/v % were prepared in 5 mL PBS containing approximately 10^6^ CFU/mL of *E. coli*. Samples were incubated during 4 h at 37 ± 1 °C and 22% ± 1% RH in a shaker.

• Effect of Temperature

Chitosan suspensions at a concentration of 0.4 wt/v % were prepared in 5 mL of PBS containing approximately 10^6^ CFU/mL of *E. coli*, and incubated during 4 h at two temperature conditions, 7 ± 1 °C and 37 ± 1 °C at 22% ± 1% RH in a shaker.

• Effect of Salt Concentration and Ionic Strength

Chitosan suspensions at concentration of 0.4 wt/v % were prepared in 5 mL of PBS containing approximately 10^6^ CFU/mL of *E. coli* and incubated during 4 h at 37 ± 1 °C and 22% ± 1% RH in a shaker. Two types of salt, NaCl and MgCl_2_ at concentrations of 0.1 M and 1.0 M were added to the PBS medium before the inoculation of bacteria and the treatment with chitosan. 

• Effect of Bacterial Species

Chitosan suspensions at concentration of 0.4 wt/v % were prepared in 5 mL of PBS containing approximately 10^6^ CFU/mL of *E. coli*, *L. innocua* or *S. aureus*, and incubated during 4 h at 37 °C and 22% ± 1% RH in a shaker. 

#### 3.2.10. Statistical Analysis

All AB tests were carried out in triplicate, and the average values with their standard deviation errors are reported. Results from the AB tests were analyzed statistically via Tukey pairwise comparisons with a confidence interval of 95% using the ANOVA-Minitab17^®^ software (trial version, Minitab Inc., State College, PA, USA). Data were normalized by re-scaling in log form.

## 4. Conclusions

In this work we have shown that chitosan in a neat discontinuous solid state can exhibit high antibacterial activity under conditions close to those of contaminated food products. This activity can be altered by factors such as pH, temperature, ionic strength, chitosan concentration, purity and bacterial species, and shown to be favored by the removal of proteins in chitosan, acidic pH conditions, and lower salt content in the medium. In addition, the presence of a solid physical form in the medium enhanced significantly the AB activity of chitosan.

Our results show the potential direct use of chitosan powder and flakes in food protection at pH values lower than chitosan p*K*_a_ (6.2–6.7). Further research on chitosan AB activity should be performed for a deeper understanding of the mechanisms and factors involved. In the scope of food protection, similar research could lead to the development of chitosan-based food packaging materials capable of inhibiting and eradicating bacteria growth.

## Figures and Tables

**Table 1:** Chitosan grades.

CS Grade ^a^	DDA (%)	MW (KDa)	PDI-	Moisture (%)	Ash (%)	mg Protein/g Chitosan	Particle Size
F-90-207 ^b^	90	207	1.7	8.1 ± 0.2	0.05 ± 0.02	8.8 ± 0.2	0.67 ± 0.40 mm
P-95-57 ^c^	95	57	2.2	9.8 ± 0.1	0.05 ± 0.01	175.7 ± 0.3	55.09 ± 43.73 μm

^a^ First letter in the nomenclature indicates F-flakes, P-powder; the first number the DDA (%) and the second one the average molecular weight (Mw, KDa). ^b^ Biolog GmbH. ^c^ Primex.

**Table 2:** Elemental analysis via EDS-SEM in chitosan grades.

Chitosan	Element										
	C	N	O	Na	Ca	Mg	S	Si	Co	Al	Cl
F-90-207	x	x	x	x	x	x		x	x	x	x
P-95-57	x	x	x	x	x	x	x		x	x	x

**Table 3:** Recovery of viable bacteria on BHI agar after exposure to 0.4 wt/v % chitosan for 4 h at 37 °C.

Chitosan Type	Survival Bacteria (log CFU/mL)			Reduction * (%)		
	Gram^−^	Gram^+^		Gram^−^	Gram^+^	
	*E. coli*	*L. innocua*	*S. aureus*	*E. coli*	*L. innocua*	*S. aureus*
Control	6.5 ± 0.6 ^a^	6.6 ± 0.5 ^a^	7.5 ± 0.8 ^a^	0.0	0.0	0.0
F-90-207	4.2 ± 0.4 ^b^	5.4 ± 0.7 ^b^	6.6 ± 0.9 ^a^	99.5	93.1	88.2
P-95-57	0.0 ^c^	0.0 ^c^	6.1 ± 0.6 ^a^	100	100	96.3

Results represent means of triplicate counts and were the same after 18 and 48 h of incubation on the agar plates, suggesting that the recovery from sub-lethal injury had not taken place. For each strain, means that do not share a letter are significantly different with a confidence level of 95% by Tukey Pairwise Comparisons. * The reduction in bacteria concentration is calculated according to Zheng & Zhu [6] $\frac{N_{1}-N_{2}}{N_{1}}\;\times \;100\%$ where N_1_ and N_2_ are the number of colony on the plates before and after treatment, respectively.
